# Risk stratification for hepatocellular cancer among patients with cirrhosis using a hepatic fat polygenic risk score

**DOI:** 10.1371/journal.pone.0282309

**Published:** 2023-02-28

**Authors:** Aaron P. Thrift, Fasiha Kanwal, Yanhong Liu, Saira Khaderi, Amit G. Singal, Jorge A. Marrero, Nicole Loo, Sumeet K. Asrani, Michelle Luster, Abeer Al-Sarraj, Jing Ning, Spiridon Tsavachidis, Xiangjun Gu, Christopher I. Amos, Hashem B. El-Serag

**Affiliations:** 1 Section of Epidemiology and Population Sciences, Department of Medicine, Baylor College of Medicine, Houston, TX, United States of America; 2 Dan L Duncan Comprehensive Cancer Center, Baylor College of Medicine, Houston, TX, United States of America; 3 Section of Gastroenterology and Hepatology, Department of Medicine, Baylor College of Medicine, Houston, TX, United States of America; 4 Center for Innovations in Quality, Effectiveness and Safety (IQuESt), Michael E DeBakey Veterans Affairs Medical Center, Houston, TX, United States of America; 5 Division of Digestive and Liver Diseases, Department of Medicine, UT Southwestern Medical Center, Dallas, Texas, United States of America; 6 Texas Liver Institute, San Antonio, Texas, United States of America; 7 Baylor University Medical Center, Dallas, Texas, United States of America; 8 Department of Biostatistics, UT MD Anderson Cancer Center, Houston, Texas, United States of America; Medizinische Fakultat der RWTH Aachen, GERMANY

## Abstract

**Background:**

Polygenic risk scores (PRS) hold the promise to refine prognostication in hepatocellular cancer (HCC). The few available HCC PRS include germline risk variants identified among individuals of mostly European ancestry, but data are lacking on the transportability of these PRS in multiethnic U.S patients with cirrhosis from multiple etiologies.

**Methods:**

We used data from 1644 patients with cirrhosis enrolled in two prospective cohort studies in the U.S. Patients were followed until HCC diagnosis, death, liver transplantation, or last study visit through June 30, 2021. The high-risk variants in *PNPLA3-MBOAT7-TM6SF2-GCKR* were combined in a PRS and we evaluated its association with HCC. Discriminatory accuracy was assessed using the C-statistic.

**Results:**

During 4,759 person-years of follow-up, 93 patients developed HCC. Mean age was 59.8 years, 68.6% were male, 27.2% Hispanic, 25.1% non-Hispanic Black, 25.7% had NAFLD, 42.1% had heavy alcohol use, and 19.5% had active HCV. HCC risk increased by 134% per unit increase in PRS (HR = 2.30; 95% CI, 1.35–3.92). Compared to cirrhosis patients in the lowest tertile of the PRS, those in the highest tertile had 2-fold higher risk of HCC (HR = 2.05; 95% CI, 1.22–3.44). The PRS alone had modest discriminatory ability (C-statistic = 0.58; 95% CI, 0.52–0.63); however, adding PRS to a predictive model with traditional HCC risk factors had a C-statistic of 0.70 (95% CI, 0.64–0.76), increasing from 0.68 without the PRS (p = 0.0012).

**Conclusions:**

Our findings suggest that PRS may enhance risk prediction for HCC in contemporary U.S. cirrhosis patients.

## Introduction

Hepatocellular cancer (HCC) is a leading cause of death worldwide [1], and is one of the few cancer types with increasing mortality rates in the United States [2]. Texas now has the highest incidence rates of HCC in the U.S., with Hispanics and African Americans disproportionally affected [3, 4]. Because cure is possible in fewer than 10% of HCC patients whose cancer is detected early and who receive liver transplantation or surgical resection, primary prevention and early detection is necessary for reducing HCC-related mortality [5].

Most cases of HCC arise in the background of cirrhosis, which is the main precursor for HCC. We recently reported that the etiology of cirrhosis has shifted into predominantly metabolic disorders from chronic viral hepatitis [6]. The absolute risk of developing HCC in cirrhosis is not uniform [7–9]; some patients progress rapidly, while others progress more slowly and a large fraction do not develop HCC. Accurate and reliable HCC risk stratification would allow targeting high risk groups for prevention including surveillance, while potentially sparing low risk groups from futile interventions. However, the variation in HCC risk, and hence risk stratification, is not reliably predicted by existing knowledge of HCC risk factors. Studies indicate an association between several inherited genetic factors and the risk of chronic liver disease, cirrhosis, and HCC but the clinical utility of genetic risk factors is unclear [10]. Polygenic risk scores (PRS) have been demonstrated to predict risk for HCC in individuals of European ancestry. In particular, studies have examined the potential for risk stratification using a PRS that included four variants previously identified in germline genetic studies (among predominantly European populations) as robustly associated with predisposition to hepatic steatosis [11–15]. This 4-SNP PRS has been shown to predict risk of HCC in patients with advanced liver disease caused by hepatitis C virus (HCV) infection [16] or non-alcoholic fatty liver disease (NAFLD) [17], and may help improve clinical risk stratification for cirrhosis progression to HCC. Despite the substantial interest in the possibility of incorporating PRS into risk stratification approaches, there are limited data on whether PRS predict risk of HCC in multiethnic U.S patients with cirrhosis from multiple etiologies.

We aimed to evaluate the performance of a PRS, including variants in *PNPLA3* (patatin-like phospholipase domain containing 3), *MBOAT7* (membrane bound O-acyltransferase domain containing 7), *TM6SF2* (transmembrane 6 superfamily member 2), and *GCKR* (glucokinase regulator), for predicting risk of developing HCC in two contemporary U.S-based multiethnic cohorts of patients with cirrhosis. This 4-SNP PRS has been previously shown to predict HCC risk in European populations, with high performance in predicting HCC risk among patients with metabolic dysfunction and NAFLD. If shown to offer independent discriminatory ability, a PRS comprising of these few SNPs may help risk stratification among an increasingly heterogenous cirrhosis population seen in contemporary hepatology clinics in the U.S.

## Materials and methods

### Study population

This project was approved by the Institutional Review Board at Baylor College of Medicine (H-40241). All participants provided written informed consent to participate in the study. We used data from 1644 patients with cirrhosis from two prospective cohort studies with genotyping: the Texas Hepatocellular Carcinoma Consortium Cohort (THCCC) [6,18] and the Houston Veterans Administration Cirrhosis Surveillance Cohort (HVASC).

The THCCC is an ongoing study that prospectively recruits patients with cirrhosis from seven institutions in four cities in Texas: Michael E. DeBakey VA Medical Center and Baylor St. Luke’s Medical Center in Houston; University of Texas Southwestern, Parkland Health & Hospital System, and Baylor University Medical Center Dallas and Baylor All Saints Fort Worth; Doctor’s Hospital at Renaissance in McAllen; and Texas Liver Institute in San Antonio. For the current analysis, we included THCCC participants enrolled between December 2016 and April 2020, with follow-up until June 30, 2021. As previously reported, cirrhosis diagnosis was based on predefined criteria for liver histology, radiology, liver elastography, or serum biomarkers [7]. We excluded patients with uncontrolled hepatic decompensation, history of HCC, or presence of non-hepatic cancer. At the time of consent, patients completed surveys including medical history and alcohol and tobacco use. We also abstracted data from the patients’ electronic medical records (EMRs) including (1) clinician-recorded diagnoses including cirrhosis etiology (e.g., HCV or hepatitis B virus [HBV] infection), complications of liver disease (ascites, varices, encephalopathy), and liver-related treatments; (2) liver imaging and liver biopsy results; and (3) laboratory data within 12 months of enrollment. Patients were scheduled for 6-month visits as part of routine clinical care and followed until HCC diagnosis, liver transplantation, or death. They received liver ultrasound, CT, or MRI for HCC surveillance per the decision of treating physician. Adherence to surveillance was high. All patients had baseline imaging as this was a criterion to be included in the cohort. Approximately 80% of our cohort underwent at least one additional HCC surveillance test. Most patients who were surveilled received ultrasound-based surveillance (81% of all patients with at least 1 surveillance test).

HVASC is a cohort of patients with cirrhosis in active HCC surveillance recruited from hepatology clinics at the Michael E. DeBakey VA Medical Center between August 2014 and December 2016, with follow-up until June 30, 2021. HVASC used similar eligibility, inclusion and exclusion criteria and recruitment procedures as those described for THCCC; indeed, most procedures for THCCC were adopted from the HVASC study. Data from THCCC and HVASC were harmonized into a common dataset using the steps described by Rolland et al. [19]. This harmonization was possible given similarities in data collection procedures and survey instruments.

### Outcome

Our primary outcome was the development of incident HCC after enrollment. HCC was defined according to the American Association for the Study of Liver Diseases criteria including histological or radiological diagnosis using characteristic appearance (arterial enhancement and delayed washout) on triple-phase CT or MRI (Liver Imaging Reporting and Data System [LI-RADS] 5) or those with suspicious lesions (LI-RADS 4) that were reviewed in multidisciplinary tumor boards and treated as HCC and adjudicated centrally. EMR reviews were conducted for all participants at 3-month intervals to capture incident HCCs, liver transplantation, and death dates. All study sites have multidisciplinary HCC tumor boards, and, for the current analysis, the date of final confirmation was used as the date of HCC diagnosis [18].

### Genotyping

For this analysis, we included a subset of patients in THCCC and HVASC that had available genotype data. Germline DNA was purified from whole-blood samples using the QIAamp DNA Blood Mini kit (Qiagen, Valencia, CA). Genotyping of germline DNA was performed on the Affymetrix Axiom Precision Medicine Research Array, which queries >902,000 common tagging SNPs. *PNPLA3 (rs738409)-MBOAT7 (rs641738)-TM6SF2 (rs58542926)-GCKR (rs1260326)* variants used in the current analysis were all directly genotyped on the array. The PRS was then calculated by summing the number of at-risk alleles (0–8), weighted by their effect size on risk of HCC in the current merged cirrhosis cohort.

### Sociodemographic and clinical risk factors

We recorded patient’s age at study enrollment, sex, and a constructed variable from self-reported race and ethnicity in the survey (non-Hispanic white, non-Hispanic Black, Hispanic, and other groups). Etiology of cirrhosis was active HCV if the patient had a positive HCV RNA test, and cured HCV if there was documentation of treatment and subsequent sustained virological response (SVR) at the time of enrollment [20]; HBV was based on a positive HBsAg [21, 22]; and alcohol-related cirrhosis based on a combination of clinician-recorded diagnosis of alcoholic liver disease and patients’ self-report of former heavy (8 or more alcoholic beverages per week for women or 15 or more alcoholic beverages per week for men) or any current use of alcohol. We used a validated survey to ascertain alcohol use that classified alcohol use status as lifetime abstention (never), former light to moderate use, former heavy use, current light to moderate use, and current heavy use. We used clinician-recorded diagnosis to define other etiologies, including autoimmune hepatitis, primary biliary cholangitis, primary sclerosing cholangitis, hereditary hemochromatosis, and Wilson’s disease. The diagnosis of NAFLD required documentation of hepatic steatosis on liver histology or imaging and the absence of HCV (active/untreated or resolved HCV), HBV, alcoholic liver disease, or other clinician documented etiologies listed above. Most (>90%) patients classified as NAFLD also had a clinician-recorded diagnosis of NAFLD in the EMR.

We defined diabetes, hypertension, and dyslipidemia based on patients’ medical history (survey or EMR) or self-reported treatment with diabetes medications (oral hypoglycemic medications or insulin), antihypertensives, or treatments for dyslipidemia (bile acid–binding resins, β-hydroxy β-methylglutaryl-CoA reductase inhibitors, nicotinic acid and fibric acid derivatives) at any time before enrollment. We defined smoking status as never, past, and current smoker based on self-report using the baseline survey. We used height and weight values at enrollment to calculate body mass index (BMI; kg/m^2^).

### Statistical analysis

Patients with cirrhosis were followed from date of enrollment in THCCC/HVASC to the development of HCC, death, liver transplantation, or end of follow up (i.e., last study visit). We estimated hazard ratios (HR) and 95% confidence intervals (95% CI) for the association between the PRS (continuous variable, and by tertiles) and incident HCC risk using Fine-Gray competing risk regression models, that accounted for the competing risks of liver transplantation and death. The discriminatory ability of the PRS was assessed using Harrell’s C-statistic and its 95% CI. We examined whether the PRS provided added discriminatory ability over demographic and clinical factors by comparing C-statistics from nested models. The demographics and clinical model was a parsimonious model with factors selected using backward stepwise selection. Potential risk factors for this model included those listed in Table 1. We also performed subgroup analyses by cirrhosis etiology and race/ethnicity to examine for potential differences or interactions in the association with the PRS. Because recent studies have shown that PNPLA3 is inversely associated with the development of diabetes among patients with NAFLD [23], we also examined the PRS separately in cirrhosis patients with and without diabetes. Finally, we examined the discriminatory ability of the PRS for predicting HCC within one year vs. ≥1 year. Analyses were conducted using SAS version 9.4 and R version 2022.07.2. All tests were two-sided and used a significance level of 0.05.

**Table 1:** Select characteristics of the study population.

Variables		N = 1,644
		N (%)
Age in years		
	Mean (SD)	59.8 (9.96)
	<55	396 (24.1)
	55–64	680 (41.4)
	≥65	568 (34.5)
Sex		
	Female	517 (31.4)
	Male	1127 (68.6)
Race/ethnicity		
	Non-Hispanic white	741 (45.1)
	Non-Hispanic Black	412 (25.1)
	Hispanic	447 (27.2)
	Other	44 (2.7)
Etiology of liver disease^1^		
	HCV active^2^	320 (19.5)
	HCV cured	469 (28.5)
	Alcoholic liver disease	251 (15.3)
	NAFLD	423 (25.7)
	HBV infection	29 (1.8)
	Autoimmune	116 (7.0)
	Other	34 (2.1)
	Missing	2 (0.1)
Alcohol drinking status		
	Never	508 (30.9)
	Current heavy	124 (7.5)
	Current but not heavy	113 (6.9)
	Past heavy	569 (34.6)
	Past not heavy	330 (20.1)
Smoking		
	Never	616 (37.5)
	Current	390 (23.7)
	Past	638 (38.8)
BMI (kg/m2)		
	Mean (SD)	30.8 (6.9)
	<25	301 (18.3)
	25–30	529 (32.2)
	> = 30	814 (49.5)
Diabetes		
	No	921 (56.0)
	Yes	723 (44.0)
Dyslipidemia		
	No	1086 (66.1)
	Yes	558 (33.9)
*PNPLA3* (rs738409)	CG/GG	919 (55.9)
*MBOAT7* (rs641738)	CT/TT	1022 (62.2)
*TM6SF2* (rs58542926)	CT/TT	213 (13.0)
*GCKR* (rs1260326)	CT/TT	877 (53.3)
PRS score (IQR)		-0.137 (-0.432, 0.087)

^1^Some patients had more than one etiological risk factor. Where possible, we relied on the primary etiology assigned by the treating clinician. Specifically, patients with HCV and excessive alcohol use were classified as patients with HCV. Alcohol-related cirrhosis was classified as the underlying risk factor when alcohol was defined as the only risk factor. NAFLD was defined as the etiology in patients without HCV, HBV, alcohol, or other etiological risk factors.

^2^HCV status was defined based on data at the time of cohort enrollment.

## Results

The mean age of the 1644 patients was 59.8 years (standard deviation, 9.96 years), 68.6% were male, 27.2% Hispanic, and 25.1% non-Hispanic Black (Table 1). Twenty-six percent of the cohort had NAFLD, 69.1% had a history of any alcohol use (including 7.5% with current heavy alcohol use and 34.6% with prior history of heavy alcohol use), 19.5% had active HCV, and 1.8% had HBV at baseline. Over 50% of cirrhosis patients had at least one risk variant in *PNPLA3*, *MBOAT7* and *GCKR*, while 13.0% had at least one risk variant in *TM6SF2*. As shown in S1 Table, the frequency of the G allele in *PNPLA3* was highest among Hispanics (65%), the frequency of the T alleles in *TM6SF2* and *MBOAT7* were similar in all races/ethnicities, and the frequency of the T allele in *GCKR* was highest in non-Hispanic whites (39%). There is some evidence of association of the PRS (tertiles) with race/ethnicity, etiology, smoking, diabetes, and dyslipidemia (S2 Table); however, the patterns were mixed.

**Fig 1:**
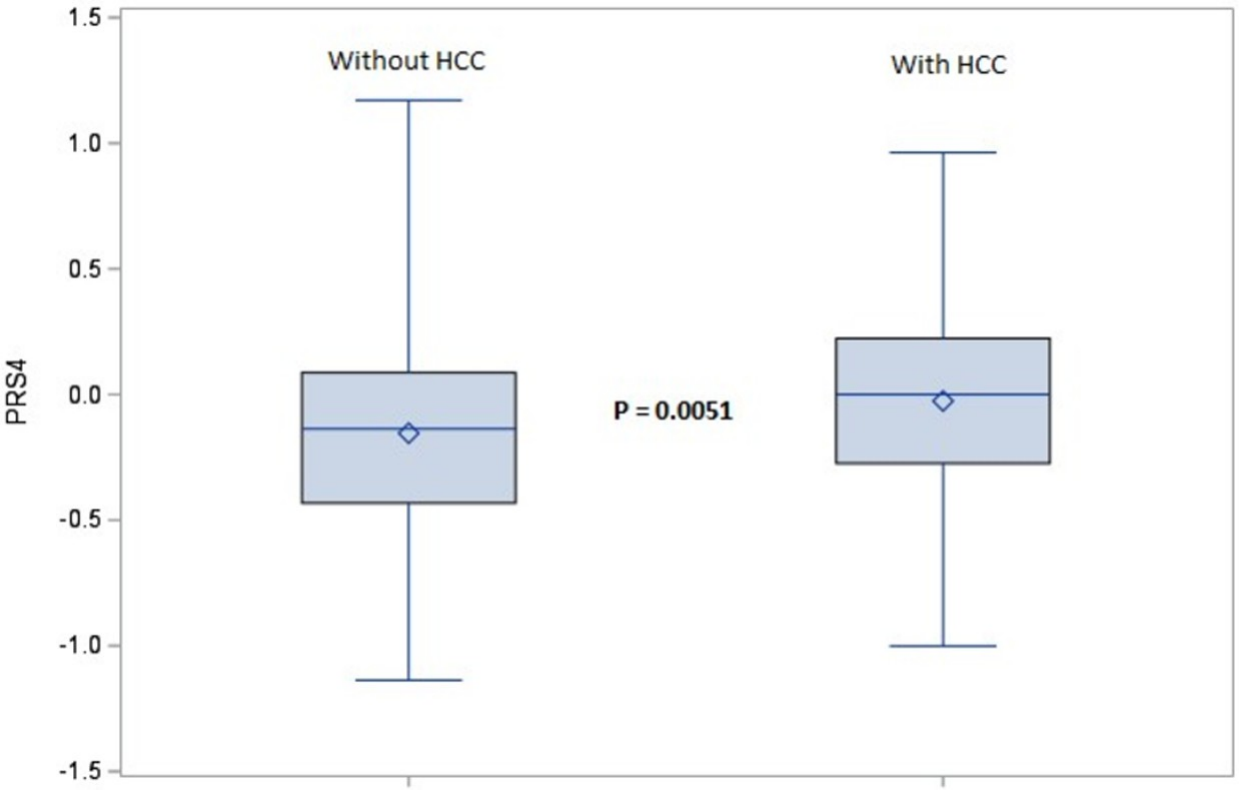
Box plot of the polygenic risk score in HCC progressors vs. non-progressors.

During 4,759 person-years of follow-up, 93 patients developed HCC. The PRS was significantly higher for patients who developed incident HCC than in those who did not progress (p = 0.0051) (Fig 1). Risk of HCC increased by 130% per one unit increase in the PRS (HR = 2.30; 95% CI, 1.35–3.92), with no attenuation in the effect estimate after adjusting for several clinical risk factors (adjusted HR = 2.34; 95% CI, 1.34–4.06). Compared to cirrhosis patients in the lowest tertile of the PRS, those in the highest tertile had 2-fold higher risk of HCC (adjusted HR = 2.05; 95% CI, 1.22–3.44) (Table 2).

**Table 2:** Association between the polygenic risk score and risk of HCC in cirrhosis patients overall and by underlying etiology and race/ethnicity.

			Unadjusted HR (95% CI)	C-statistic (95% CI)
**Overall**		T2 vs. T1	1.48 (0.86–2.55)	0.58 (0.52–0.63)
		T3 vs. T1	2.05 (1.22–3.44)	
**Underlying etiology**				
	NAFLD	T2 vs. T1	0.69 (0.13–3.78)	0.58 (0.46–0.70)
		T3 vs. T1	1.93 (0.58–6.44)	
	Non-NAFLD	T2 vs. T1	1.57 (0.87–2.82)	0.59 (0.53–0.64)
		T3 vs. T1	2.16 (1.22–3.84)	
	HCV	T2 vs. T1	1.51 (0.80–2.86)	0.57 (0.50–0.64)
		T3 vs. T1	1.84 (0.95–3.59)	
	Alcoholic liver disease	T2 vs. T1	0.88 (0.15–5.27)	0.62 (0.48–0.76)
		T3 vs. T1	2.36 (0.62–9.04)	
**Race/ethnicity**				
	Hispanic	T2 vs. T1	1.48 (0.43–5.05)	0.55 (0.46–0.64)
		T3 vs. T1	1.75 (0.56–5.46)	
	Non-Hispanic white	T2 vs. T1	1.01 (0.44–2.30)	0.59 (0.50–0.68)
		T3 vs. T1	2.09 (1.04–4.18)	
	Non-Hispanic Black	T2 vs. T1	2.44 (0.72–8.31)	0.57 (0.49–0.65)
		T3 vs. T1	2.53 (0.69–9.21)	
**Diabetes**				
	No	T2 vs. T1	2.02 (1.00–4.07)	0.57 (0.50–0.64)
		T3 vs. T1	1.86 (0.89–3.88)	
	Yes	T2 vs. T1	0.90 (0.37–2.21)	0.59 (0.51–0.67)
		T3 vs. T1	2.16 (1.03–4.51)	

T1, tertile 1; T2, tertile 2; T3, tertile 3.

The model with PRS alone had modest discriminatory ability for HCC with a C-statistic of 0.58 (95% CI, 0.52–0.63). A model that included demographic and clinical risk factors (age, sex, alcohol drinking status, and dyslipidemia, derived from backward selection) had a C-statistic of 0.68 (95% CI, 0.63–0.74). The model that included these factors in addition to the PRS had a C-statistic of 0.70 (95% CI, 0.64–0.76), a statistically significant increase in discriminatory ability (0.70 vs. 0.68; p = 0.0012).

The PRS was strongly and similarly associated with HCC risk in subgroups of cirrhosis patients according to cirrhosis etiology or race/ethnicity, but the 95% CI were wide due to small numbers of HCC in each of these stratified analyses (Table 2). Since the PRS was developed previously for predicting HCC progression in patients with NAFLD, we compared its performance in patients with NAFLD-cirrhosis vs. non-NAFLD-cirrhosis. In NAFLD-cirrhosis patients, those in the highest tertile of the PRS had around 2-fold higher risk of HCC compared to those in the lowest tertile (HR = 1.93; 95% CI, 0.58–6.44) and the PRS had a C-statistic of 0.58 for discriminating between HCC progressors and non-progressors. Likewise, in non-NAFLD-cirrhosis patients, carrying more risk variants was associated with over 2-fold high risk for HCC (highest vs. lowest tertile, HR = 2.16; 95% CI, 1.22–3.84) and the PRS demonstrated had modest discriminatory ability for HCC (C-statistic = 0.59). The association of the PRS with HCC risk and its discriminatory ability was similar among cirrhosis patients with and without diabetes as well as HCC development within and beyond one year (Table 2).

## Discussion

Using data from two contemporary prospective cohorts of cirrhosis patients, we examined whether a weighted 4-SNP PRS estimating an individual’s susceptibility to accumulating liver fat would predict incident HCC development. We confirmed that the 4-SNP PRS was associated with the future risk of HCC, and that this association was independent of traditional clinical risk factors (e.g., alcohol drinking status, dyslipidemia). In contrast to prior studies, which included only individuals of European ancestry [16, 17], ours included a multiethnic cohort of which 27% were Hispanic and 25% non-Hispanic Black. Our study provides evidence that the PRS is associated with HCC risk in all racial/ethnic subgroups of cirrhosis patients. Furthermore, the PRS was associated with HCC risk in the setting of viral, alcohol and NAFLD-related cirrhosis. Risk stratification and surveillance for HCC in cirrhosis patients may therefore be improved using data from this PRS based on four validated risk variants.

Our PRS included four variants previously identified in germline genetic studies among predominantly European populations as robustly associated with predisposition to hepatic steatosis [11–15]. Although using a different mechanism, carrying the risk allele (shown in S1 Table) results in increased susceptibility to accumulation of fat within intracellular lipid droplets in hepatocytes. Two prior analyses that examined the same 4-SNP PRS for HCC prediction that we did also demonstrate its discriminatory ability in specific liver disease patients. Bianco et al. [17] focused on predicting HCC risk among patients with NAFLD or members of the general population with metabolic risk factors but no advanced liver fibrosis or cirrhosis. The authors showed that genetic predisposition (as determined by having a higher PRS) was associated with increased HCC risk in individuals with NAFLD/dysmetabolism, and that the PRS had modest discriminatory ability (AUROC, 0.64 in NAFLD patients and 0.69 and 0.70 in individuals in the general population with BMI ≥30 or diabetes, respectively). The association between the PRS and HCC was partly, but not completely, mediated by the promotion of severe fibrosis [17]. Degasperi et al. [16] examined the association between the PRS and risk of HCC in patients with HCV-related cirrhosis seen in an Italian tertiary care center. They found that the PRS was associated with the incidence of de novo HCC, and that this association was independent of classical risk factors (i.e., sex, diabetes, albumin) and the severity of liver disease. We observed similar associations in our multiethnic contemporary cohort of patients with cirrhosis from multiple etiologies. Specifically, the magnitude of the association of the PRS with HCC risk and its discriminatory ability were similar among patients regardless of their cirrhosis etiology (NAFLD, HCV, alcohol related) or race/ethnicity.

Although the PRS was strongly associated with the risk of HCC, with those in the highest tertile having over 2-fold higher risk of HCC compared to those in the lowest tertile of the PRS, the PRS alone had a moderate accuracy to predict HCC (C-statistic, 0.58). These data are consistent with prior studies of PRS for cancer risk stratification. However, we found that combining the PRS into a single model with established HCC risk factors may improve risk stratification for HCC. Specifically, the model discrimination improved from 0.68 to 0.70 with the addition of the PRS (p = 0.0012). Similar findings of improved, albeit modest, discriminatory ability of the PRS over a clinical risk factor only model have been reported previously [16]. Additional improvement in discriminatory ability may be achieved by adding additional SNPs to the PRS; however, cost-effectiveness of such approach remains to be tested. A limited number of SNPs could have its benefits in terms of cost.

Strengths of our study include the multicenter, prospective cohort study design using standardized methods across centers, with a broad range of patient ages, multiple racial and ethnic groups, both sexes, cirrhosis from multiple etiologies, and minimal loss to follow-up. We used a previously derived PRS for HCC that had been validated in multiple studies, but only among majority European populations. We showed for the first time the potential utility of this PRS for HCC risk stratification among multiple races and ethnicities. However, our study has several limitations. Despite following strict protocols for follow-up, with structured EMR reviews to ascertain outcomes, we may have missed HCC events in some patients who sought care outside of the health care systems where recruitment for the study took place. Although we used validated surveys for alcohol use and smoking, we cannot exclude measurement bias. NAFLD diagnosis was a constructed variable defined based on the absence of other active risk factors (HCV or alcohol) but not based on demonstration of fatty liver disease, which is difficult to do in cirrhosis, potentially resulting in misclassification bias. However, most patients classified as NAFLD had a physician-documented diagnosis of NAFLD and had at least one metabolic risk factor, providing support to our definition. We likely underestimated the presence of NAFLD because we did not account for the possibility that some patients with viral hepatitis may also have metabolic dysfunction–associated fatty liver disease [24]. Due to the relatively small number of HCC progressors, the 95% CI were wide due in each of the stratified analyses, limiting our ability to identify true differences between subgroups. We also examined a limited number of potential predictors due to the smaller number of HCCs. It is possible that the addition of other clinical factors and biomarkers would enhance further the discriminatory ability of the comprehensive risk model. Finally, because we followed patients in hepatology specialty clinics, our findings may not be generalizable to broader populations of patients with cirrhosis.

In conclusion, in this cohort of contemporary cirrhosis patients in the U.S., we confirmed that validated 4-SNP PRS estimating susceptibility to accumulation of liver fat is associated with risk of HCC. Furthermore, the PRS may offer discriminatory advantage over clinical and lifestyle factors for HCC risk stratification. Additional research is needed to define a comprehensive risk model for predicting HCC progression in cirrhosis patients that includes PRS which accounts for the patient’s underlying disease etiology as well as genetic differences between racial and ethnic subgroups.

## Supporting information

S1 Table Risk variant allele frequency in patients with cirrhosis, overall and by race/ethnicity. (DOCX)

S2 Table Risk variant allele frequency in patients with cirrhosis, overall and by race/ethnicity. (DOCX)
