# Supplementary material for: Risk stratification for hepatocellular cancer among patients with cirrhosis using a hepatic fat polygenic risk score
Source: PLoS One. 2023 Feb 28;18(2):e0282309. doi: 10.1371/journal.pone.0282309 (PMC9974109; doi:10.1371/journal.pone.0282309)
Supplement: S1 Table — (DOCX) [file pone.0282309.s001.docx]

**S1 Table.** Risk variant allele frequency in patients with cirrhosis, overall and by race/ethnicity.

|  | **All subjects** | **Non-Hispanic White** | **Non-Hispanic Black** | **Hispanic** |
| --- | --- | --- | --- | --- |
| rs1260326 (*GCKR*; T allele) | | | |  |
| MAF | 0.33 | 0.39 | 0.16 | 0.28 |
| rs58542926 (*TM6SF2*; T allele) | | | |  |
| MAF | 0.07 | 0.08 | 0.05 | 0.04 |
| rs641738 (*MBOAT7*; T allele) | | | |  |
| MAF | 0.38 | 0.40 | 0.35 | 0.38 |
| rs738409 (*PNPLA3*; G allele) | | | |  |
| MAF | 0.35 | 0.42 | 0.19 | 0.65 |
