# Supplementary material for: Risk stratification for hepatocellular cancer among patients with cirrhosis using a hepatic fat polygenic risk score
Source: PLoS One. 2023 Feb 28;18(2):e0282309. doi: 10.1371/journal.pone.0282309 (PMC9974109; doi:10.1371/journal.pone.0282309)
Supplement: S2 Table — (DOCX) [file pone.0282309.s002.docx]

**S2 Table.** Risk variant allele frequency in patients with cirrhosis, overall and by race/ethnicity.

| Variables |  | T1  N=563 | T2  N=553 | T3  N=528 | p-value |
| --- | --- | --- | --- | --- | --- |
| Age in years |  |  |  |  |  |
|  | Mean (SD) | 60.4 (9.98) | 59.9 (10.42) | 59.2 (9.41) | 0.12 |
| Sex |  |  |  |  | 0.09 |
|  | Female | 193 (34.3) | 156 (28.2) | 168 (31.8) |  |
|  | Male | 370 (65.7) | 397 (71.8) | 360 (68.2) |  |
| Race/ethnicity |  |  |  |  | <0.001 |
|  | Non-Hispanic white | 342 (60.8) | 203 (36.7) | 196 (37.1) |  |
|  | Non-Hispanic Black | 93 (16.5) | 207 (37.4) | 112 (21.2) |  |
|  | Hispanic | 110 (19.5) | 134 (24.2) | 203 (38.5) |  |
|  | Other | 18 (3.2) | 9 (1.6) | 17 (3.2) |  |
| Etiology of liver disease^1^ |  |  |  |  | <0.001 |
|  | HCV active^2^ | 97 (17.2) | 135 (24.4) | 88 (16. 7) |  |
|  | HCV cured | 175 (31.1) | 180 (32.5) | 114 (21.6) |  |
|  | Alcoholic liver disease | 88 (15.6) | 67 (12.1) | 96 (18.2) |  |
|  | NAFLD | 147 (26.1) | 108 (19.5) | 168 (31.8) |  |
|  | HBV infection | 14 (2.5) | 10 (1.8) | 5 (1.0) |  |
|  | Autoimmune | 35 (6.2) | 45 (8.1) | 36 (6.8) |  |
|  | Other | 7 (1.2) | 8 (1.5) | 19 (3.6) |  |
|  | Missing | 0 (0) | 0 (0) | 2 (0.4) |  |
| Alcohol drinking status |  |  |  |  | 0.37 |
|  | Never | 185 (32.9) | 159 (28.8) | 164 (31.1) |  |
|  | Current heavy | 37 (6.6) | 46 (8.3) | 41 (7.8) |  |
|  | Current but not heavy | 29 (5.2) | 47 (8.5) | 37 (7.0) |  |
|  | Past heavy | 190 (33.8) | 196 (35.4) | 183 (34.7) |  |
|  | Past not heavy | 122 (21.7) | 105 (19.0) | 103 (19.5) |  |
| Smoking |  |  |  |  | <0.001 |
|  | Never | 205 (36.4) | 199 (36.0) | 212 (40.2) |  |
|  | Current | 128 (22.7) | 164 (29.7) | 98 (18.6) |  |
|  | Past | 230 (40.9) | 190 (34.4) | 218 (41.3) |  |
| BMI (kg/m2) |  |  |  |  |  |
|  | Mean (SD) | 30.8 (7.0) | 30.3 (6.4) | 31.3 (7.1) | 0.09 |
|  | <25 | 102 (18.1) | 107 (19.4) | 92 (17.4) | 0.21 |
|  | 25-30 | 185 (32.9) | 183 (33.19) | 149 (28.2) |  |
|  | >=30 | 276 (49.0) | 263 (47.6) | 287 (54.4) |  |
| Diabetes |  |  |  |  | 0.02 |
|  | No | 332 (59.0) | 319 (57.7) | 270 (51.1) |  |
|  | Yes | 231 (41.0) | 234 (42.3) | 258 (48.9) |  |
| Dyslipidemia |  |  |  |  | 0.01 |
|  | No | 356 (63.2) | 393 (71.1) | 337 (63.8) |  |
|  | Yes | 207 (36.8) | 160 (28.9) | 191 (36.2) |  |
